# Comparison of Three-Dimensional Micro-CT Angiography of Cervical Spinal Cord between Two Contrast Agents

**DOI:** 10.1155/2019/5215923

**Published:** 2019-04-11

**Authors:** Yapu Liu, Qi Liu, Zhou Yang, Junyu Lin, Xiuhua Wu, Zucheng Huang, Junhao Liu, Rong Li, Zhiping Huang, Xiaoliang Wu, Qingan Zhu

**Affiliations:** ^1^Department of Spinal Surgery, Nanfang Hospital, Southern Medical University, Guangzhou, China; ^2^Department of Spinal Surgery, Second Affiliated Hospital of Luohe Medical College, Luohe, China

## Abstract

**Purpose:**

Barium sulfate and lead oxide are commonly used for angiographic studies, but there is no report on the comparison of two contrast agents in angiography of cervical spinal cord. This study was aimed to compare the microvascular architecture of cervical spinal cord in rats after angiography with the barium sulfate agent to the lead oxide agent.

**Methods:**

Twelve adult Sprague-Dawley rats were randomly divided into the barium sulfate group (*n*=6) and the lead oxide group (*n*=6). Each rat was perfused under the same protocol using either two contrast agents. The angiography was evaluated with the vascular number at different ranks. The cervical spinal cord samples were scanned using micro-CT with low resolution and high resolution. The microvascular parameters, including ratio of vascular volume to tissue volume (VV/TV), vascular number (V.N), diameter (V.Dm), separation (V.Sp), connectivity density (Conn.D), structure model index (SMI), percentage, and volume of vessels at different diameters were measured.

**Results:**

The perfusion was better in the barium sulfate group, with more blood vessel trees of rank II and III visible compared to the lead oxide group. Low-resolution micro-CT analysis showed no difference in microvascular parameters except SMI between the two groups. High-resolution micro-CT analysis results showed that V.N and Conn.D of barium sulfate group were 60% and 290% more than those of the lead oxide group; however, V.Sp was 41% less than the lead oxide group. The percentage of vessels with diameter of 10 *μ*m and 20 *μ*m, and the volume of vessels with diameter of less than 100 *μ*m was higher in the barium sulfate group than in the lead oxide group. The SMI index in the barium sulfate group was higher than that in the lead oxide group at both low resolution and high resolution.

**Conclusions:**

Compared with lead oxide, barium sulfate is more suitable for perfusion of cervical spinal cord microvessels, and cheap and nontoxic with high resolution.

## 1. Introduction

Complete vascular architecture is an important basis for maintaining neurological homeostasis in the central nervous system. Vascular injury contributes to the further development of neural network damage [1]. Considering that the pathological process of the disease is usually associated with microvascular damage, it is fundamentally required to visualize the spatial distribution of vascular architecture [2]. However, in order to assess the involvement of vasculature in pathological processes or to measure the benefits of therapeutic strategies, the vascular network must be accurately quantified and visualized.

Different techniques can be used to observe vasculature. Tissue sectioning is a traditional method widely used as a golden standard for detecting spinal microvascular changes [3]. However, tissue sections disrupt the three-dimensional structural integrity of the spinal cord specimen and provide only a two-dimensional (2D) image of the vascular system, which provides incomplete spatial information of the vessel. Computed tomography combined with angiography has recently been developed to visualize 3D vascular structure in experimental and clinical studies [4]. Rodents are often used for experimental studies of spinal vascular changes because of the similarity of blood supply systems compared to humans [4, 5]. Spinal vascular structure can be reconstructed by intravascular perfusion of spinal cord with micro-CT scanning.

Barium sulfate and lead oxide contrast agents are commonly used in cadaver-based angiographic studies. Lead oxide was used for visualizing the vascular networks [6, 7] but resulted in metal artifacts on CT images at high-resolution scanning. Barium sulfate is an alternative contrast agent without obvious metal artifacts on CT images. However, the two contrast agents have not been compared in angiography of cervical spinal cord. Therefore, the purpose of this study is to compare the microvascular architecture of cervical spinal cord in rats after angiography with barium sulfate and lead oxide agents.

## 2. Materials and Methods

### 2.1. Animals and Study Design

A total of 12 adult Sprague-Dawley rats were divided into the barium sulfate group (*n*=6) and the lead oxide group (*n*=6). Each animal was perfused with the barium sulfate or the lead oxide agents. The cervical spinal cord samples were subjected to the micro-CT scan at low and high resolution, respectively. The microvascular architecture of spinal cord was evaluated between two groups.

All animals were purchased from the Southern Medical University Experimental Animal Centre (Guangzhou, China). The animal procedures were conducted in accordance with the National Institutes of Health guidelines for the care and use of experimental animals and were approved by the Southern Medical University Institutional Animal Care and Use Committee.

### 2.2. Perfusion Agent Preparation

Barium sulfate gelatin preparation: 20 g of barium sulfate (Sachtleben, Germany) and 5 g of gelatin (Aladdin, China) were added into 100 ml of physiological saline, and the mixture was heated at 37°C and continuously stirred until the gelatin was completely dissolved and uniformly mixed, which took about 2 hours.

Lead oxide gelatin preparation: 20 g of lead oxide (Pb_3_O_4_ red lead; Aladdin, China) and 5 g of gelatin (Aladdin, China) were added into 100 ml of physiological saline, heating the mixture at 37°C and stirring continuously until the gelatin was completely dissolved and uniformly mixed.

### 2.3. Vascular Perfusion

The perfusion methods we have used refer to previous reports [6, 8]. All rats were deeply anesthetized by intraperitoneal injection of Beuthanasia (200 mg/kg). Fully sedated rats were immediately performed an abdominothoracic incision to expose the heart, liver, and intestines. A 22-gauge obtuse cannula connecting the pressure gauge was inserted into the aorta via the left ventricle and then perfused with heparinized saline. After decoloration of the liver and the presence of limpid drainage fluid, the circulatory blood was completely expelled. Thereafter, 300 ml of 4% paraformaldehyde was perfused under the same pressure. Effectual perfusion was characterized by hardening of the body and tail. The perfusion contrast agent was continuously infused into the aortic cannula until the hepatic lobules and intestinal wall blood vessels were completely white or red. Constant pressure was used to ensure consistent perfusion. After the obtuse cannula was removed, the thoracic aorta was ligated and the perfused animals were moved to a refrigerator at 4°C overnight. We retained the nerve roots when harvesting the spinal cord, making it easy to reference location during prescanning. The cervical spinal cords with C5 in the centre were harvested and fixed with 4% paraformaldehyde for another 24 h.

### 2.4. Rank of Vessels

We evaluated the angiographic effects of contrast agents by calculating the vascular number of the perfused cervical spinal cords from C3 to C7 in different ranks [9]. Radially distributed large vessels were defined as rank I blood vessels, which were branched into rank II blood vessels, and rank III blood vessels were branches of rank II blood vessels. Vascular number was counted from C3 to C7 segments using the analysis software (ACD See v5.0, ACD Systems, Canada).

### 2.5. Micro-CT Scanning

The harvested cervical spinal cord sample was fixed to a small cystosepiment and aligned in the plastic tube (U80810, outer diameter 20 mm, inner diameter 18.5 mm, height 65 mm) for micro-CT scanning (*μ*CT80, Scanco Medical, AG, Switzerland).

The sequence parameters of low resolution were an isotropic voxel size of 20 *μ*m in a 20.5mm field of view (55 kv, 145 *μ*A, 8 W, integration time 300 ms, source-to-sample distance 59013 *μ*m, source-to-detector distance 271513 *μ*m, averaged two times), 500 projections per rotation and 338 slice image acquisition required approximately 45 min per spinal cord. The sequence parameters of high resolution were an isotropic voxel size of 8 *μ*m in a 20.5 mm field of view (55 kv, 145 *μ*A, 8 W, integration time 300 ms, source-to-sample distance 59013 *μ*m, source-to-detector distance 271513 *μ*m, averaged two times), 1000 projections per rotation and 846 slice image acquisition required approximately 2.5 h per spinal cord. Each sample was embedded in the centre of a sponge, which was subsequently laid in a rotating focus X-ray tube for 360° scanning to obtain the tomograms. To keep the consistency of scanning, we retained the nerve roots when harvesting the spinal cord, making it easy for reference location during prescanning and setting the same number of scan layers. In the present study, we referred to the report of Perrien et al. [10] for analytical parameters. Vascular indexes of cervical spinal cord were ratio of vascular volume to tissue volume (VV/TV), vascular number (V.N), diameter (V.Dm), separation (V.Sp), connectivity density (Conn.D), structure model index (SMI), percentage, volume of vessels with different diameter, etc.

### 2.6. Statistical Analysis

Statistical analysis was performed using Excel (Office 2007, Microsoft, WA). All measurements were expressed as mean ± standard error of mean. The Student's *t*-test was used to compare vascular indexes measured by micro-CT scanning, and a *P* value <0.05 was considered to be statistically significant.

## 3. Results

### 3.1. General Aspects

**Figure 1:**
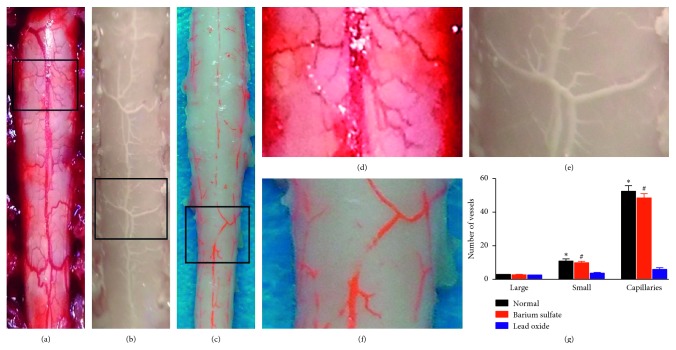
Pictures of cervical spinal cord vessels *in vivo* and *in vitro*. (a) The posterior middle artery of the cord and its branch of cervical spinal cord of rats *in vivo*. (b) After perfusion with the barium sulfate. (c) After perfusion with the lead oxide. Local magnification of typical vascular conditions ((d) partial detail from (a), (e) partial detail from (b), and (f) partial detail from (c)). (g) There were more blood vessel trees of ranks II and III visible in the barium sulfate group compared to the control group. ^*∗*^denotes *P* < 0.05 between the normal and lead oxide groups. ^*#*^denotes *P* < 0.05 between the barium sulfate and lead oxide groups.

In the barium sulfate group, the contrast agent was evenly distributed on the surface of the liver, intestine, and cervical spinal cord, and the blood vessel trees were clearly visible (Figures 1(b) and 1(e)), with vascular numbers of rank I 2.80 ± 0.20, rank II 10.00 ± 0.84, and rank III 49.00 ± 2.59 (Figure 1(g)). In the lead oxide perfusion group, the contrast agent distribution was not uniform on the surface of liver, intestine, and spinal cord after lead oxide perfusion, and most vascular trees were not distinguishable (Figures 1(c) and 1(f)), with vascular numbers of rank I 2.32 ± 0.08, rank II 3.80 ± 0.47, and rank III 6.00 ± 1.05. (Figure 1(g)). There were no difference in vascular numbers of rank I between the two groups, and vascular numbers of rank II and rank III in the barium sulfate group were higher than the barium sulfate group (*P*=0.001).

### 3.2. Low-Resolution Micro-CT Analysis Results

**Figure 2:**
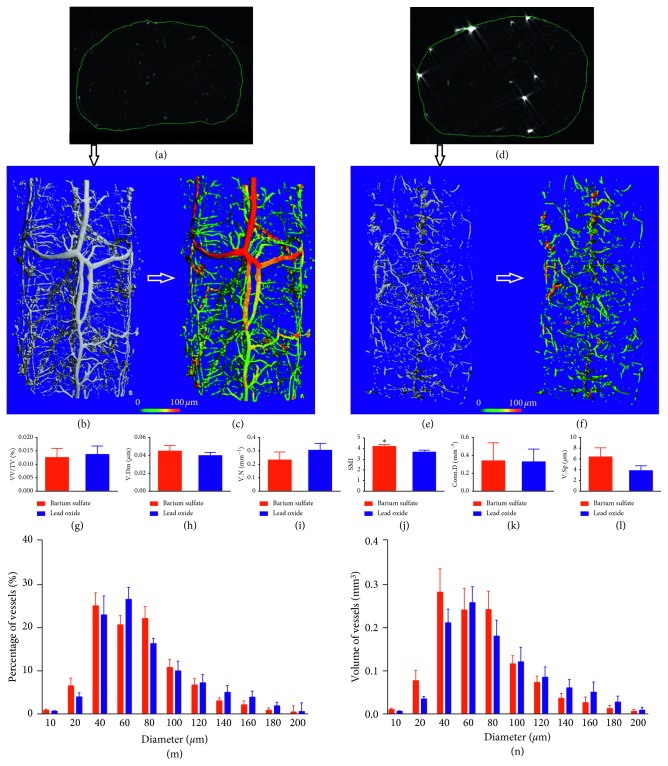
Vasculars of the cervical spinal cord at low-resolution micro-CT scanning. The spinal cord contour is defined around the outer edge of the spinal cord (a, d). The resulting mask was applied in combination with a grayscale threshold and a three-dimensional Gaussian noise filter to segment the perfused vasculature from the total volume (b, e). The vascular diameter was calculated using a distance transform method (c, f). The bar graphs showing VV/TV, V.Dm, V.N, SMI, Conn.D, V.Sp, percentage, and volume of vessels (g–n). There was no difference in VV/TV, V.Dm, V.N, Conn.D, V.Sp, percentage, or volume of vessels with different diameters between the two groups, except significant difference in SMI.

**Figure 3:**
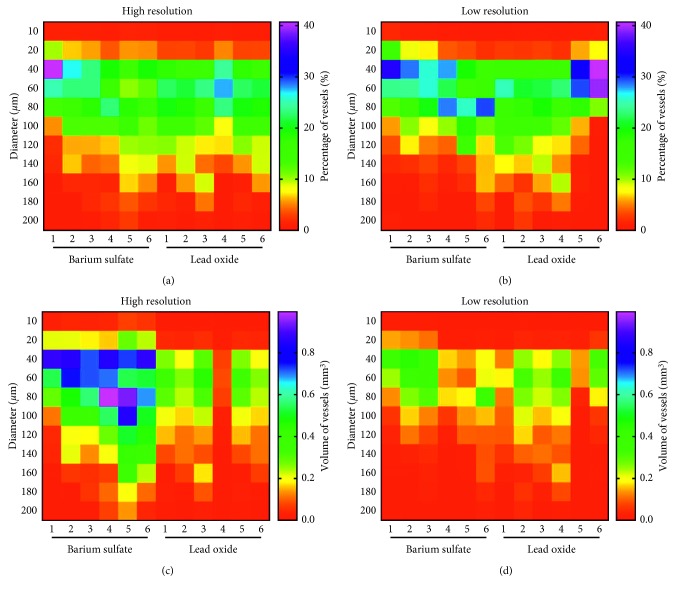
Hot map of vessels percentage and volume with different diameters. (a) Vessels percentage with different diameters in high-resolution scanning. (b) Vessels percentage with different diameters in low-resolution scanning. (c) Vessels volume with different diameters in high-resolution scanning. (d) Vessels volume with different diameters in low-resolution scanning. The numbers 1, 2, 3, 4, 5, and 6 represent the number of rats in each group.

The spinal cord volume was defined using a single contour around the outer edge of the spinal cord (Figures 2(a) and 2(d). The resulting mask was applied in combination with a grayscale threshold and a 3D Gaussian noise filter to segment the perfused vasculature from the total volume (Figures 2(b) and 2(e)) and used a distance transform method to calculate the thickness (Figures 2(c) and 2(f)). The 3D reconstruction analysis results showed no difference in VV/TV (Figure 2(g)), V.Dm (Figure 2(h)), V.N (Figure 2(i)), Conn.D (Figure 2(k)), V.Sp (Figure 2(l)), percentage (Figures 2(m) and 3(b)), and volume of vessels (Figures 2(n) and 3(d)) at different diameters between the two groups. However, SMI (Figure 2(j)) in the barium sulfate group was 4.21 ± 0.05, which was higher than that of 3.68 ± 0.08 in the lead oxide group.

### 3.3. High-Resolution Micro-CT Analysis Results

**Figure 4:**
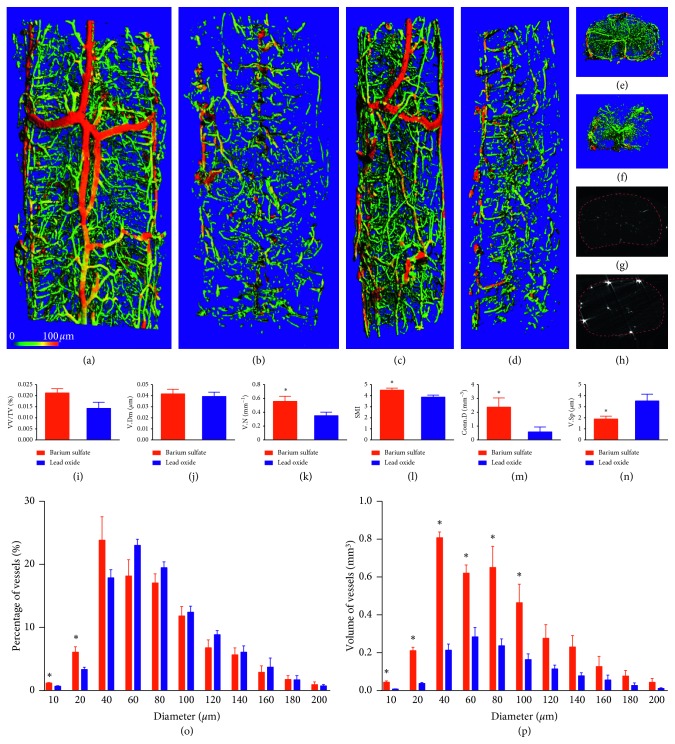
Vasculars of the cervical spinal cord at high-resolution micro-CT scanning. The intact view of 3D microvasculature following the barium sulfate (a) and lead oxide (b) perfusion. Lateral view of 3D microvasculature following the barium sulfate (c) and lead oxide (d) perfusion. Cross-sectional 3D reconstruction of 100 slices in the middle part of the scanned spinal cord following the barium sulfate (e) and lead oxide (g) perfusion. 2D micro-CT image of the cervical spinal cord microvasculature in the barium sulfate group (f) and lead oxide group (h). The bar graphs showing VV/TV, V.Dm, V.N, SMI, Conn.D, V.Sp, percentage, and volume of vessels (i–p). There was no significant difference in VV/TV and V.Dm between the two groups. V.N, SMI, and Conn.D of barium sulfate group were higher than those of the lead oxide group, with V.Sp less than that of lead oxide group. ^*∗*^denotes *P* < 0.05 between the barium sulfate and lead oxide groups.

Two-dimensional micro-CT image of the cervical spinal cord microvasculature in the barium sulfate group is shown in Figure 4(f). The contrast agent was evenly distributed in the spinal cord, and the microstructure of blood vessels in the parenchyma was clearly visible. However, in the lead oxide group, a large amount of artifact interference was seen in the two-dimensional image (Figure 4(h)). In the 3D imaging, the anterior and posterior spinal artery in the barium sulfate group could be seen; also, the branch arteries were clearly visible and continued well from the front view (Figure 4(a)), lateral view (Figure 4(c)), and cross section view (Figure 4(e)). In the lead oxide group, the microvasculature was poor in continuity and shape from the front view (Figure 4(b)), lateral view (Figure 4(d), and cross section view (Figure 4(g)). The 3D reconstruction analysis results showed that the V.N (Figure 4(k)), SMI (Figure 4(l)), and Conn.D (Figure 4(m)) in the barium sulfate group were 60%, 290%, and 27% greater than those in the lead oxide group, respectively, while the V.Sp in the barium sulfate group was 41% less than that in the lead oxide group (Figure 4(n)). In the hot map of vessels (Figure 3), each box represents the volume percentage or vessel volume of corresponding vessel diameter with low or high resolution, which is a further intuitive refinement of Figures 2(m), 2(n), 4(o), and 4(p). In the barium sulfate group, the percentage of vessels with diameter of 10 *μ*m and 20 *μ*m (Figures 4(o) and 3(a)) and the volume of vessels with a diameter of less than 100 *μ*m (Figure 4(p)) were higher than those in the lead oxide group (Figure 3(c)). However, there was no difference in VV/TV or V.Dm between the two groups (Figures 4(i) and 4(j)).

## 4. Discussion

In this study, we recommend that barium sulfate is more suitable for perfusion imaging and 3D reconstruction of cervical spinal cord microvessels, which is of high resolution, cheap, and nontoxic. High-resolution micro-CT analysis results showed that the percentage of vessels with diameter of 10 *μ*m and 20 *μ*m and the volume of vessels with diameter of less than 100 *μ*m in the barium sulfate group were higher than those in the lead oxide group. Low-resolution micro-CT analysis results showed no difference in microvascular parameters except SMI between the two groups. Lead oxide appeared as metal artifacts in the cervical spinal cord micro-CT scanning, affecting the analysis of vascular microstructure. This study laid the foundation for the further application of barium sulfate for microvascular perfusion.

Cheng et al. [11] and Long et al. [12] reported the use of the lead oxide combined with micro-CT scanning for cervical vascular changes in a rat chronic compression model, but the microvascular perfusion was relatively poor. The present study further showed that the microvascular perfusion of cervical spinal cord was not ideal with the lead oxide at low-resolution or high-resolution micro-CT scanning. In addition, there were obvious metal artifacts on the micro-CT images after the lead oxide perfusion, which might be caused by accumulation of lead oxide in relatively large vascular or vascular herniation. We considered that the artifacts of lead oxide seriously affected the accuracy of 3D reconstruction. Moreover, lead oxide is an environmental pollutant, which can cause toxic effects in multiple organ systems [13, 14]. The use of lead oxide requires a strict personal protective equipment protocol, and any residues and contaminated equipment require specialist disposal [7]. Although lead oxide is widely used, it is highly toxic and therefore suboptimal in terms of safety.

Kingston et al. [6] compared the angiography effects of barium sulfate and lead oxide in lower limb vessels of rats. The scanning voxel size was 295 *μ*m with low resolution and 59 *μ*m with high resolution. It was found to have less deviation and less estimated variance with the barium sulfate than lead oxide at high-resolution micro-CT scanning. However, there was no significant difference between the number of branching generations. The smallest vessel diameter visible on the rat arterial network 3D reconstructions was 70 *μ*m. We thought the voxel size with high-resolution scan was still too thick. In the present study, the scanning voxel size was adjusted to 20 *μ*m to observe the perfusion effect in cervical spinal cord of rats and test any difference between the lead oxide and barium sulfate groups. Then, the further comparison was performed with 8 *μ*m high resolution. The present study further confirmed that the perfusion of barium sulfate resulted in a clear cervical spinal cord angioarchitecture and presented microvessels at high-resolution scanning. In high-resolution micro-CT scans, the display of microvessels after perfusion of barium sulfate was better than that of lead oxide. Barium sulfate showed better microvascular display at 10 *μ*m and 20 *μ*m at high resolution, and was better than lead oxide, and the overall continuity of reconstructed blood vessels was superior to that of lead oxide. Barium sulfate provided a better indication of the microvascular structure.

Microfil is a silicon containing lead chromate that perfuses well into the soft tissues. Some studies have used Microfil for perfusion [15–20]. After Microfil perfusion, the microvascular structure of the spinal cord is relatively clear. However, the Microfil contrast material is expensive, especially when used in large quantities for experiments on rats or other large animals. Moreover, studies have shown that the minimal blood vessels following Microfil perfusion is about 10 *μ*m [21–23], and the minimum blood vessels following barium sulfate perfusion is almost the same, so we excluded this contrast material in our study from the perspective of research economics and effects. Other studies have shown that barium sulfate infusion provided a more suitable choice for vessel images than Microfil [24, 25]. Blery et al. [26] have made a lot of tests using these two contrast agents. Barium sulfate has been demonstrated to give better results as in the literatures [25, 27, 28]. Blery also tested a number of mixtures with different gelatin or barium sulfate percentages. However, previous studies [25–27] suggest the optimal technique was a warmed mixture of barium sulfate via heart perfusion techniques. The present study adopted a similar protocol, i.e., perfusion at temperature of 37°C and produced satisfactory microvessels of spinal cord in rats.

In recent years, there have been reports on the use of synchrotron radiation devices to observe spinal cord microvasculature [2, 29], but there are many drawbacks unsolved, such as huge equipment, high cost, and incapability of miniaturization of devices, which cannot be widely promoted at present. Nowadays, contrast medium perfusion combined with micro-CT scanning reconstruction is still the most widely used method for three-dimensional imaging of spinal cord microvasculature.

One limitation of this study is that the current scanning condition was based on the literature [6, 10] and our experience [30, 31] with the Scanco equipment. The scanning condition may not be ideal for the lead-agent.

## 5. Conclusions

Taking together, we successfully compared the effects of lead oxide and barium sulfate in the perfusion of cervical spinal cord microangiography in rats. At high-resolution micro-CT scanning, the percentage of vessels with diameter of 10 *μ*m and 20 *μ*m and the volume of vessels with diameter of less than 100 *μ*m in the barium sulfate group were higher than those in the lead oxide group. The metal artifact of lead oxide after micro-CT scanning affected the analysis of microstructure parameters of blood vessels. Barium sulfate, as a more suitable perfusion agent with high resolution, low cost, and nontoxicity, combined with micro-CT scanning can be used as a preclinical significance research tool for monitoring 3D morphological structure of spinal cord microvessels.

## Data Availability

The data used to support the findings of this study are included within the article.
